# Feasibility of Equivalent Dipole Models for Electroencephalogram-Based Brain Computer Interfaces

**DOI:** 10.3390/brainsci7090118

**Published:** 2017-09-15

**Authors:** Paul H. Schimpf

**Affiliations:** Department of Computer Science, Eastern Washington University, Cheney, WA 99004, USA; pschimpf@ewu.edu; Tel.: +1-509-359-6908

**Keywords:** brain–computer interface, electroencephalogram, equivalent source model

## Abstract

This article examines the localization errors of equivalent dipolar sources inverted from the surface electroencephalogram in order to determine the feasibility of using their location as classification parameters for non-invasive brain computer interfaces. Inverse localization errors are examined for two head models: a model represented by four concentric spheres and a realistic model based on medical imagery. It is shown that the spherical model results in localization ambiguity such that a number of dipolar sources, with different azimuths and varying orientations, provide a near match to the electroencephalogram of the best equivalent source. No such ambiguity exists for the elevation of inverted sources, indicating that for spherical head models, only the elevation of inverted sources (and not the azimuth) can be expected to provide meaningful classification parameters for brain–computer interfaces. In a realistic head model, all three parameters of the inverted source location are found to be reliable, providing a more robust set of parameters. In both cases, the residual error hypersurfaces demonstrate local minima, indicating that a search for the best-matching sources should be global. Source localization error vs. signal-to-noise ratio is also demonstrated for both head models.

## 1. Introduction

The vast majority of non-invasive brain computer interfaces (BCI) based on the electroencephalogram (EEG) focus on temporal events such as sensorimotor rhythms and evoked potentials [1]. Few attempts have been made to make use of source localization other than by strategic location of the EEG recording electrodes. This is perhaps due to the fact that temporal information in the EEG is well understood, reproducible, and not overly challenging to extract in terms of the required real-time processing power. In contrast, inverting the EEG to localize brain activity is known to be an ill-posed problem [2], and is highly underdetermined when linearized with a lead-field approach [3]. While there are a number of algorithms to circumvent this problem, they tend to be compute-intensive and might represent some challenges for real-time computation in a portable system. In any event, few attempts to make use of equivalent source localization for EEG-based BCI have been published.

Qin, L. et al. reported on a pilot study for BCI based on equivalent source location using off-line EEG data, wherein subjects were asked to imagine right or left hand movement based on a visual cue [4]. The time window and frequency band of the event-related desynchronization (ERD) [5] were hand-selected from the data, pre-processed via independent component analysis [6], and inverted to a source map using a regularized weighted minimum norm [7]. A single best-fit equivalent dipole was also found using the Nelder–Mead Simplex method [8]. A three concentric sphere head model was used to represent the domain [9]. All currents within 80% of the largest were summed over each hemisphere, and if the sum on the ipsilateral side was larger than the contralateral side, the source mapping method was judged to have correctly classified the imagined hand movement. If the single best-fit equivalent dipole was located in the ipsilateral hemisphere of the brain, the equivalent dipole approach was judged to have correctly classified the imagined hand movement. Single-trial classification accuracy of 78.9% (source map) and 80.6% (equivalent dipole) were reported on a single test subject. Because the time and frequency data were hand-selected, no detection algorithm was discussed and thus no information was available regarding false positives. Nevertheless, the results indicated promise for the idea of using the localization of equivalent dipoles from EEG for BCI. No information was included on processing time, although it was stated that the approach could be implemented in an on-line BCI application with little alteration. While the noise subspace was estimated and discarded, and the source signal-to-noise ratio (SNR) was reported to be better than the scalp SNR, no numerical estimates of the SNR were reported.

A follow-up study [10] expanded the analysis to two equivalent dipoles. Manual selection of the time windows was again applied and Nelder–Mead Simplex was again used for the search. The method was judged to have correctly classified the imagined hand movement if the stronger dipole was located in the ipsilateral hemisphere. An average classification rate across four subjects was 80%, a promising result but not higher than the preliminary study using a single dipole.

It is important to note that the equivalent dipole locations, orientations, and amplitudes with this approach are not intended to represent an accurate model of brain activity. They instead represent an amalgam of brain activity as represented by a few dipolar sources. When a lead-field approach is used the forward problem is represented by a linear system as [3]:

(1) Ls=v

where **L** is the (*m* × 3*n*) lead-field, or forward transfer, matrix, **s** is the (3*n* × 1) vector of dipolar source amplitudes, with each dipolar source represented by three orthogonal components of the dipole moment, and **v** is the (*m* × 1) vector of EEG samples. In the most general case, the number of unknown source amplitudes (3*n*) is far greater than the number of EEG measurements (*m*), representing a severely underdetermined problem that is also ill-posed [2]. There are a number of algorithms that select from the infinity of solutions via various assumptions. When the number of non-zero sources is known to be, or can be restricted to be, some number less than 3*n*, the problem becomes underdetermined, with a single solution in the least-squares sense:

(2) $$s_{q}={\left(L_{q}^{T}L_{q}\right)}^{-1}L_{q}^{T}v$$

where **q** indicates some subset of the *n* candidate sources along with the corresponding columns of **L**. One must then search for the subset **q** that minimizes the residual

(3) $$\underset{q}{min}{\left\|L_{q}s_{q}-v\right\|}^{2}$$

The question in this case is not the accuracy to which the fitted dipoles represent actual brain activity, but rather whether the location of the best fitting dipoles are useful as classification parameters in a BCI algorithm. This will depend on a subject’s ability, perhaps with training, to control the equivalent dipole locations. Because of the ill-posed nature of the problem, a reasonable precursor question is the extent to which the actual best fitting sources can be found given the head model being used. That is the question investigated here.

## 2. Materials and Methods

### 2.1. Head Models

The reproducibility of a single known equivalent dipole in the face of noisy EEG measurements was investigated in both a four-shell spherical head model [11] and a realistic head model.

**Figure 1:**
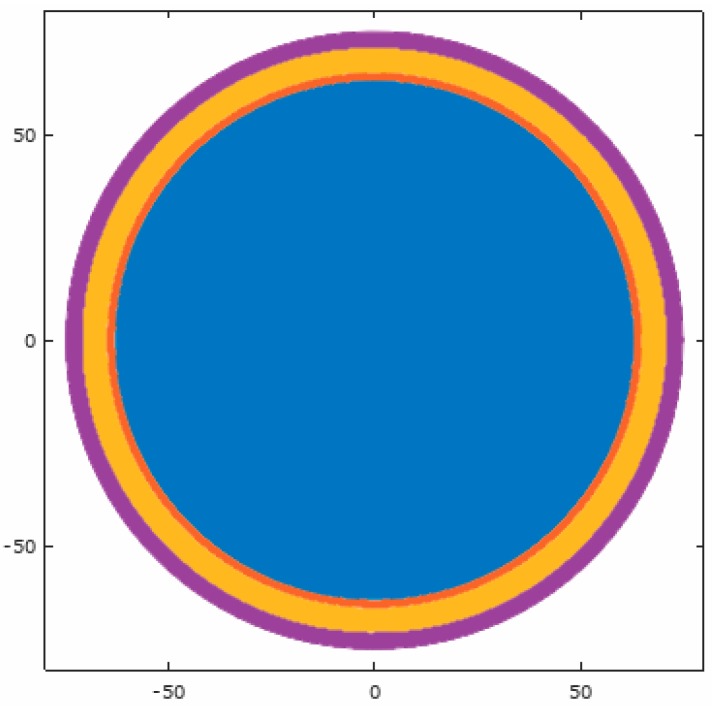
Cross-section through the center of the spherical model.

The four-shell model has been used in several other studies (e.g., [12,13,14,15]) and is composed of concentric spheres of varying conductivity representing the brain, cerebral-spinal fluid (CSF), skull, and scalp, as shown in Figure 1. The origin of the model is the center of the spheres, and the radii of the tissue boundaries were set at 63, 65, 71, and 75 mm respectively. The modeled conductivities of the layers were 0.33, 1.0, 0.042, and 0.33 S/m, respectively [16,17,18,19].

**Figure 2:**
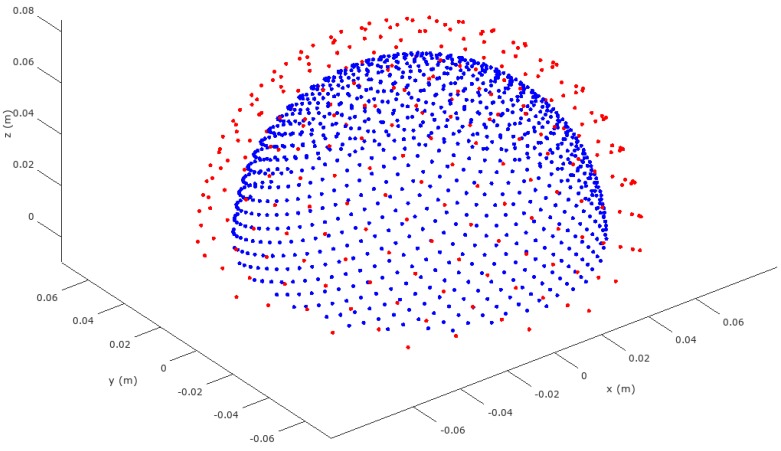
Candidate source and EEG electrodes in spherical head model (upper hemisphere, sources in blue, EEG electrodes in red).

1000 candidate source locations were distributed pseudo-uniformly [20] over the upper hemisphere of the cortical surface, with an approximate spacing of 4.8 mm. 256 EEG electrodes were likewise distributed over the upper hemisphere of the scalp surface. The locations of candidate sources and electrodes are shown in Figure 2. The forward problem for each candidate source was obtained from the analytic solution in [11] and used to form a 256 × 3000 lead-field, with three orthogonal dipole moment directions per candidate source location.

**Figure 3:**
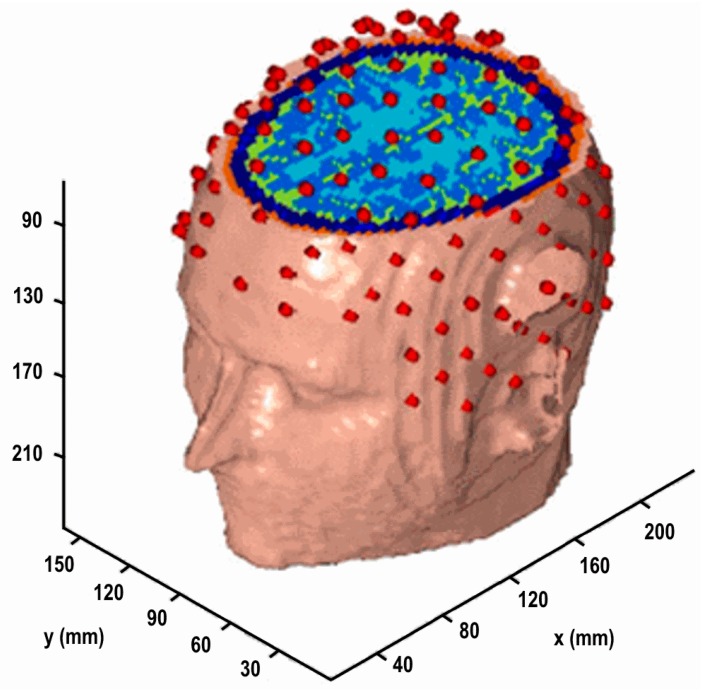
Realistic head model with cutaway showing internal tissues. EEG electrodes in red.

The realistic head model is based on classified magnetic resonance images of a human head. The tissues were classified according to the method discussed in [21]. The model contained 11 tissue types with resistivities obtained from [16]. EEG observation locations were obtained from the 82 points of an extended EEG 10–20 layout, visually interpolated with an additional 63 points, resulting in 145 EEG channels. The candidate source space consisted of 3035 locations sampled from the cortical surface at a resolution of 4.0 × 4.0 × 3.2 mm, resulting in a lead field matrix of dimension 145 × 9105, with three orthogonal source directions per location. The lead-field matrix was calculated using the finite element method [15,22]. This model is illustrated in Figure 3. This model, along with a finite element solver for non-conforming meshes, is available from the author at [23].

### 2.2. Source Localization Error

As discussed in the introduction, the primary question is the theoretical extent to which equivalent dipoles can be reliably located from the EEG given the mathematically ill-posed nature of the inverse problem. This question was investigated using random trials on the two head models. For each model, ground-truth dipoles were generated at random source locations, with random orientations. The forward model of Equation (1) was solved for EEG measurements and normally distributed noise was added. The best fitting dipole to the noisy EEG was then found by performing an exhaustive search for the location that minimized the residual error in Equation (3). The mean and standard deviation of the Euclidean distance between the actual and inverted dipole locations was then plotted against signal-to-noise (SNR) ratio. Surface plots of the residual error in Equation (3) over all candidate source locations were also examined for the presence of local minima, in order to provide insight as to whether a global search algorithm is required for this approach.

## 3. Results and Discussion

### 3.1. Spherical Head Model

**Figure 4:**
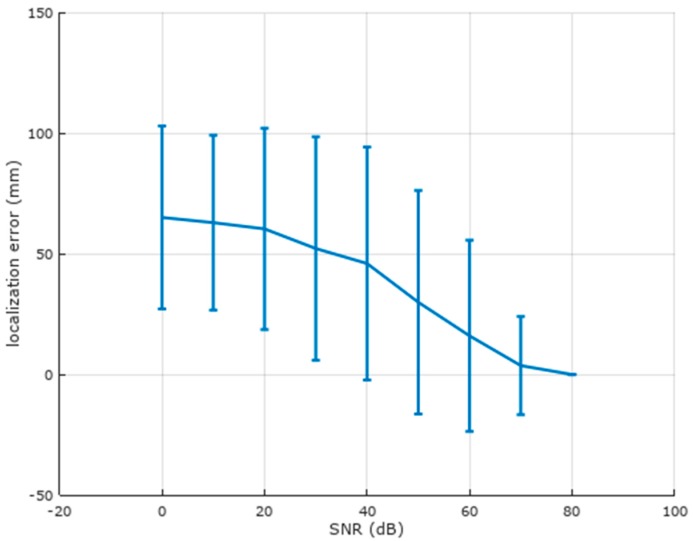
Localization error vs. signal-to-noise (SNR) ratio for the spherical model. Mean and standard deviation are shown for 250 randomized trials at each SNR. Localization is poor below 70 dB (the radius of the cortical sphere is 63 mm).

Figure 4 illustrates localization error vs. SNR for the spherical head model for 250 randomized trials at each SNR. The possible range of localization error depends on the location of the ground truth source in the hemisphere of possibilities (see Figure 2) which was selected randomly for each trial. Figure 4 shows that the localization degrades dramatically for SNRs lower than 70 dB, which is an unrealistically large SNR for single trial EEG recordings. The compression of the standard deviation at lower SNRs is explained by the fact that the domain is finite and inverted sources are restricted to the cortical surface, which creates an upper limit to the possible localization error. As the noise level increases (lower SNR), the mean localization approaches an asymptote given by the worst case scenario of a completely random guess. As the center (mean) of the histogram increases, the width of the histogram is compressed because of the upper limit to the error. Thus, when the noise is low, both the mean and standard deviation are small (the localization is reliable), both grow with the noise level until the width of the histogram begins to encroach on the upper limit on error, which causes the histogram width to then shrink somewhat as the mean continues to approach the worst case scenario. As an aid to interpretation of the trend in Figure 4, a completely random guess as to source location when the true location is at the apex (north pole) of the cortical surface has a mean of 53 mm and a standard deviation of 25 mm. When the true location is anywhere on the equator, the mean error of a completely random guess is 85 mm with a standard deviation of 25 mm.

**Figure 5:**
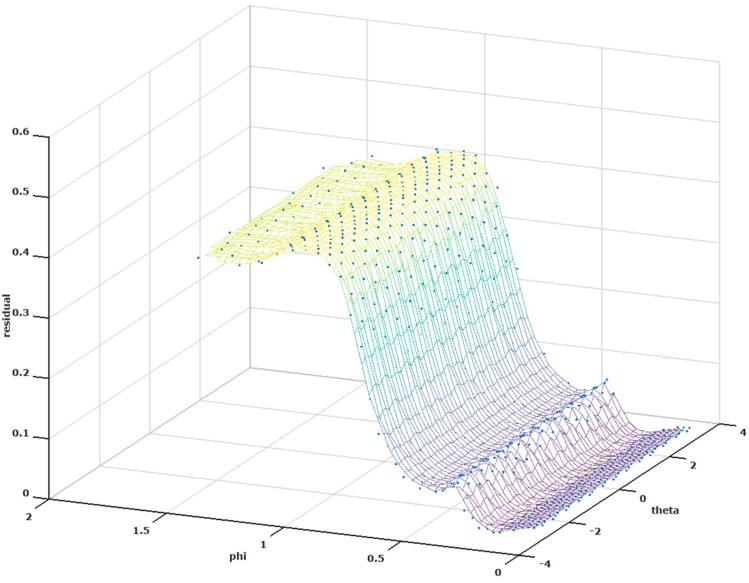
Residual at all candidate sources for an illustrative noise-free case. All good candidate sources (low residual) reside at the same elevation (phi), with a range of azimuths (theta) that covers the entire 2π radians.

A fundamental problem is that constraining the inverse to an underdetermined system does not avoid the ill-posed nature, and small perturbations in the measurement can produce widely different localizations. In this case, however, the localization ambiguity occurs only for the azimuth of the inverted source location. This is illustrated for noise-free data in Figure 5, which plots the residual from Equation (3) for all candidate sources against the azimuth (theta) and elevation (phi) of the source. All candidate sources reside at the same radius (63 mm) in this model. This shows that there are a number of good fits to the EEG data (i.e., leaving a small residual) that are at different azimuths, but only one elevation.

**Figure 6:**
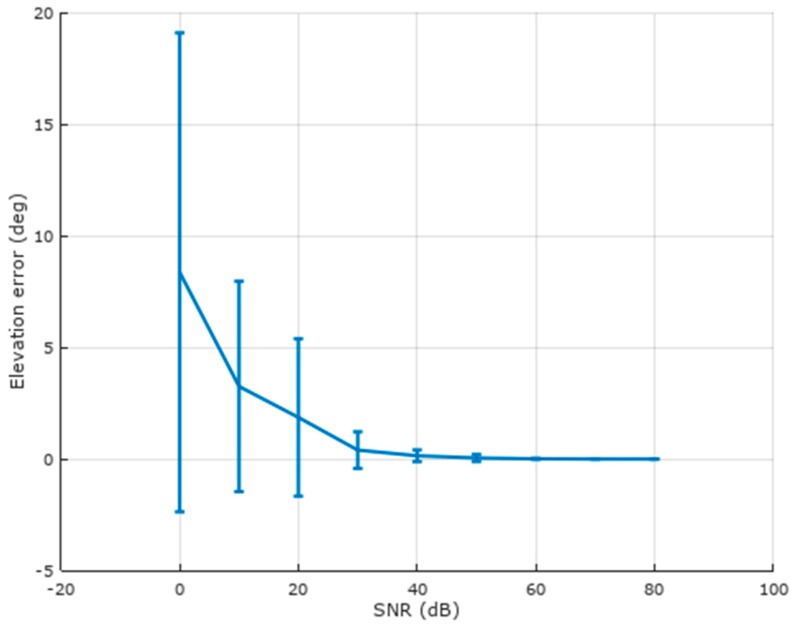
Elevation error vs. signal-to-noise (SNR) ratio for the spherical model. Mean and standard deviation are shown for 250 randomized trials at each SNR. The elevation range for candidate sources is 0 to 90 degrees.

Figure 6 further illustrates this point by plotting the elevation error vs. SNR, which performs well over a much larger SNR range than source location.

**Figure 7:**
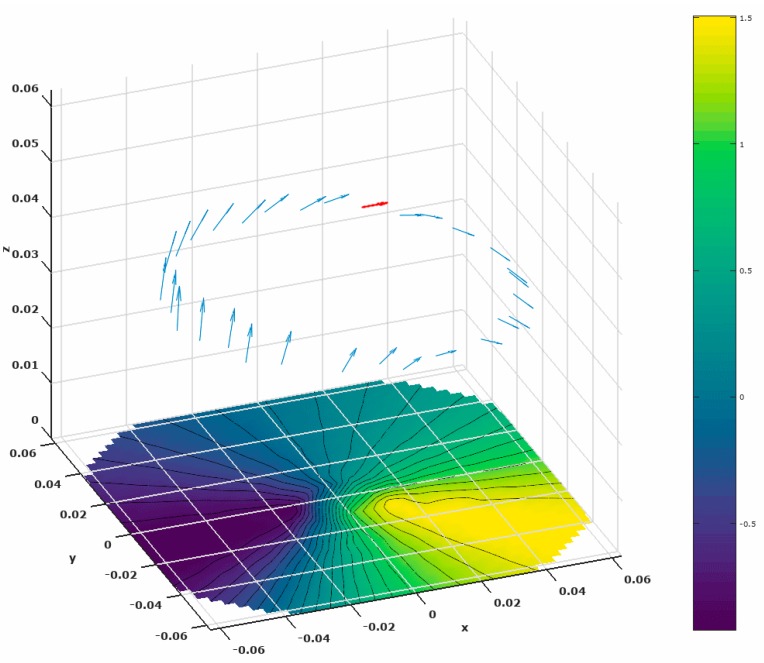
Dipolar sources that produce an Electroencephalogram (EEG) within 1% of the EEG produced by the true source (red). The surface EEG produced by the true source is plotted at the bottom of the figure.

Figure 7 provides additional insight into the azimuth ambiguity. It plots the location and moment of a ground truth dipole source (red) along with the locations and moments of all candidate sources that provide an EEG residual within 1% (blue). The EEG is plotted underneath the dipoles as an aid to understanding how these different dipoles could produce the same EEG on the surface of the model. This shows that there are a number of good candidate sources that can reproduce the surface EEG, distributed around the entire circumference of the cortex, all with differing orientations. Thus only the elevation can provide reliable information in this case. It is the spherical symmetry of the model that produces this ambiguity in the azimuth of the inverted sources.

Taken together, Figure 5, Figure 6 and Figure 7 indicate that only the elevations of inverted sources are reliably invertible from the EEG when a spherical head model is used. Because the azimuth of the location is unreliable, including it in the parameter set could confound a BCI algorithm when a spherical head model is used.

### 3.2. Realistic Head Model

**Figure 8:**
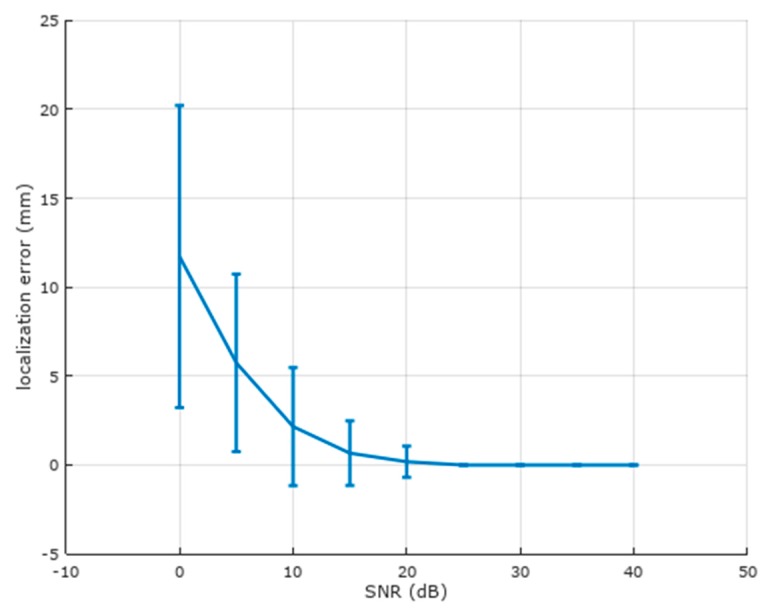
Localization error vs. signal-to-noise (SNR) ratio for the realistic head model. Mean and standard deviation are shown for 250 randomized trials at each SNR. Localization is much better than the spherical head model at lower SNRs.

Figure 8 illustrates localization error vs. SNR for the realistic head model. It performs much better over a range of SNRs than the spherical head model, with reasonable results down to 10 dB. The use of a realistic head model thus provides at least three reasonable BCI parameters per inverted source: x, y, and z, or equivalently, r, theta, and phi.

### 3.3. Local vs. Global Minima

**Figure 9:**
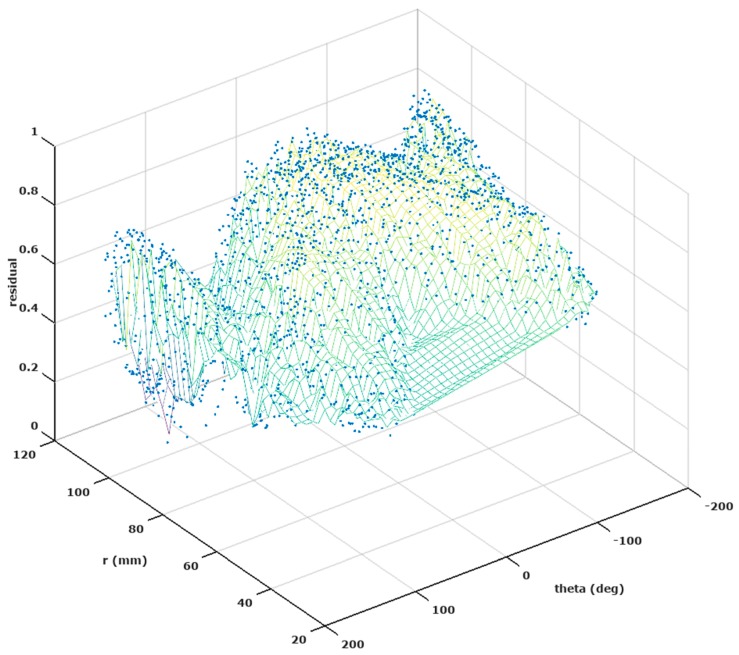
Residual at all candidate sources for an illustrative noise-free case in the realistic head model.

For the spherical head model, Figure 5 also illustrates local minima in the residual error surface that would trap a localized search for the minimum residual. In the realistic head model, the origin for candidate sources was placed in the Cerebellum in order to compare approximately to the coordinate system of the spherical model. A difference is that candidate sources in the realistic model do not all fall at the same radius. The residual error hypersurface thus has three dimensions and cannot be shown in its entirety on a single plot. Figure 9 shows the projection of a residual hypersurface onto the azimuth (theta) and radial directions, indicating the presence of local minima for the realistic head model as well. Thus a localized search for the best-fitting dipoles for either model cannot be expected to be reliable, and a global search strategy should be used.

A simple approach to a global search is to exhaustively compute the residual for all possibilities, which has a computational complexity of *O*(*n^p^*), where *n* is the number of candidate sources (one-third the number columns in the lead-field) and *p* is the number of sources allowed in the solution. This can become quickly intractable as *p* increases. On a Core i3-4360 CPU (3.7 GHz, Intel Inc., Santa Clara, CA, USA) with 16 GB of DDR3 RAM (Crucial Inc., Gretna, LA, USA) running Octave 4.2.1 ([24]) under Microsoft Windows 7 (Microsoft Corp., Redmond, WA USA), this search required less than 0.1 s for *p* = 1 on the realistic head model. A value of *p* = 2 required 202 s. The pseudo-inverses for different column subsets of the lead-field are trivially parallelizable. Thus *p* = 2 is within the range of real-time performance if a speedup on the order of 200 can be achieved. This is currently under investigation using GPU parallelization.

### 3.4. Summary and Applicability

The results above show that the following should be taken into account when inverting equivalent dipole locations from the EEG:

(a) When a spherical head model is used, the inverted azimuth of the dipole is unreliable in the face of even modest amounts of noise, although the elevation of the dipole is reliable.
(b) All components of the location are reasonably reliable when a realistic head model is used.
(c) The residual cost function for locating the dipole exhibits local minima, and thus a global search strategy should be used.

For an EEG-based BCI, the performance cannot therefore be expected to be optimal if a spherical head model is used with the full inverted dipole location, or when using a localized search strategy.

It should be noted that it is not necessarily the case that a realistic head model for BCI be subject-specific. Given the somewhat limited success of using a spherical model [4,10], along with these findings on location ambiguity, there is good reason to expect that any realistic model, such as [25], would improve BCI performance without needing to be subject-specific.

The findings here are specific to source localization from the EEG. Similar concerns may or may not exist for source localization from the magnetoencephalogram (MEG), which is related to the EEG problem via the Biot–Savart law [15]:

(4) $$B=\frac{\mu_{0}}{4\pi }\int \frac{J\times \hat{r}}{r^{2}}dv$$

The use of a vector measurement (**B**) and the fact that **J** represents both volume and source currents may mitigate the source localization ambiguities found with the EEG, which is something not investigated here.

It is worth noting that other modalities provide more direct opportunities to characterize the location of brain activity. As an imaging technology, functional magnetic resonance imaging (fMRI) [26] provides spatial information directly. Functional near-infrared spectroscopy (fNIRS) [27] provides some opportunities for obtaining spatial information through the strategic placement of emitter–detector pairs. The fNIRS technology appears promising and may well prove to supplant EEG as a common BCI approach when inexpensive fNIRS headsets become available as is the case for EEG (e.g., [28]).

## 4. Conclusions

This work shows that equivalent dipole approaches to EEG-based BCI produce ambiguous results in source azimuth when a spherical head model is used, and can thus be expected to provide reliable information only in terms of source elevation. It also shows that a realistic head model does not suffer such localization ambiguity, providing a more robust set of parameters for potential BCI classification. It further shows that the EEG residual hypersurface demonstrates local minima, indicating that a search for best-fitting dipolar sources should be global.
